# Analyzing the Effectiveness of Different Delivery Methods for Remineralization Agents in Pediatric Dental Health: A Systematic Review

**DOI:** 10.7759/cureus.76577

**Published:** 2024-12-29

**Authors:** Bahija Basheer, Abdulmalik A Alqahtani, Asem Abdullah Alowairdhi, Sultan Nuri Alohali

**Affiliations:** 1 College of Dentistry, King Saud bin Abdulaziz University for Health Sciences, Riyadh, SAU; 2 Faculty of Dentistry, King Abdullah International Medical Research Center, Riyadh, SAU; 3 Department of Dentistry, Ministry of National Guard Health Affairs, Riyadh, SAU

**Keywords:** dental caries prevention, dental foams, dental gels, dental sprays, “enamel remineralization”, remineralization agents, remineralization delivery methods

## Abstract

Pediatric dental health is critically impacted by enamel demineralization and early-stage caries, with remineralization therapies playing a vital role in preventing progression. This systematic review evaluates the effectiveness of various delivery methods for remineralization agents in pediatric patients aged 3-15 years, focusing on varnishes, gels, foams, and sprays. Studies were included if they were randomized controlled trials (RCTs), cohort studies, or case-control studies involving pediatric patients with early-stage caries or enamel demineralization, while studies with non-pediatric populations, unrelated treatments, or significant methodological flaws were excluded. A comprehensive search across PubMed, Cochrane, Google Scholar, and Web of Science using keywords like "pediatric," "fluoride varnish," "CPP-ACP," and "enamel remineralization" identified 10 RCTs with 743 participants from countries such as Germany, Italy, Turkey, China, Thailand, Iran, and Brazil. Data extraction followed a standardized protocol, and quality was assessed using the Cochrane Risk of Bias 2 tool. Results showed that fluoride varnishes were the most effective delivery method for high-risk caries populations due to their prolonged retention and sustained fluoride release, while sprays and foams emerged as promising alternatives for behaviorally challenging patients because of their ease of application and better compliance. Gels demonstrated mixed results, indicating that their efficacy might depend on application frequency and patient adherence. The findings also underscore the importance of cultural and regional factors in the acceptance of certain treatment methods. Additionally, long-term studies are needed to evaluate the sustained effects of sprays and foams in pediatric populations. This review highlights the critical role of delivery methods in the success of remineralization therapies, emphasizing that fluoride varnishes remain the gold standard, while sprays and foams are viable alternatives for specific patient needs. Tailoring treatments to individual patient requirements is essential for optimizing outcomes in pediatric dental health, particularly in addressing diverse patient behaviors and treatment environments.

## Introduction and background

Dental caries remains one of the most prevalent chronic diseases among children worldwide, posing a significant public health challenge [1]. Despite advances in preventive and therapeutic strategies, caries management in pediatric patients continues to demand innovative approaches, especially during the early stages of enamel demineralization [2]. Early-stage caries, characterized by non-cavitated lesions, represent a critical window for intervention, as timely remineralization can reverse the progression of demineralization, preserving tooth structure and minimizing the need for invasive treatments [3]. Addressing this challenge involves not only identifying effective remineralization agents but also determining the optimal delivery methods to maximize clinical outcomes [4].

Remineralization agents such as fluoride-based products, casein phosphopeptides (CPP), calcium phosphates, and other biomimetic materials have shown promise in promoting enamel repair and preventing caries progression [5]. These agents work by replenishing lost minerals in the enamel matrix, strengthening its structure, and reducing the susceptibility to acid attacks from cariogenic bacteria [6]. While the efficacy of these agents has been well-documented, the mode of delivery (as varnishes, gels, foams, sprays, or other forms) can influence their clinical effectiveness, ease of application, and patient compliance [7]. The pediatric population presents unique challenges in the management of dental caries. Children aged 3-15 years are particularly vulnerable to caries due to developing oral hygiene habits [8], frequent exposure to cariogenic diets, and the physiological characteristics of primary and newly erupted permanent teeth, which are less mineralized than adult teeth [9]. Additionally, pediatric patients often have behavioral and psychological considerations that impact their acceptance of dental procedures [10]. Thus, selecting a delivery method that balances efficacy with practicality and patient comfort is critical for achieving favorable outcomes.

Different delivery methods offer distinct advantages and limitations in clinical practice. Varnishes, for instance, are widely used due to their ease of application and prolonged contact time with enamel, which enhances fluoride uptake [11]. Gels and foams, often applied in dental trays, can provide high concentrations of remineralizing agents but may pose challenges in younger children who have difficulty tolerating prolonged tray placement [12]. Sprays, while less commonly studied, represent a promising alternative for their simplicity and minimal invasiveness. Each method also varies in its ability to distribute the agent uniformly, maintain therapeutic concentrations, and penetrate enamel lesions effectively. Understanding these differences is essential for tailoring interventions to the specific needs of pediatric patients. For instance, in high-caries-risk populations, a more intensive delivery method, such as varnish or gel, may be preferable. Conversely, in children with behavioral challenges or dental anxiety, less invasive options like sprays or quick-application foams may improve compliance and treatment acceptance. However, the existing literature lacks comprehensive comparisons of these delivery methods, particularly in the context of their relative effectiveness in pediatric populations.

What is the relative effectiveness of different delivery methods of remineralization agents in enhancing enamel remineralization, preventing caries progression, and improving oral health outcomes in pediatric patients? While numerous studies have evaluated the efficacy of individual remineralization agents, relatively few have focused on comparing their delivery methods in pediatric populations. The existing evidence is fragmented, often limited to single-center studies or narrow comparisons that do not account for variations in patient demographics, dental conditions, or application protocols [13,14]. Moreover, the methodological quality of these studies varies, with many failing to include appropriate control groups or standardized outcome measures. This lack of robust evidence complicates the task of establishing clinical guidelines for the use of remineralization agents in children.

Furthermore, the heterogeneity in study designs, including variations in age groups, intervention protocols, and outcome measures, has made it challenging to synthesize findings into actionable insights. For example, while fluoride varnishes are a mainstay in pediatric caries prevention, their relative performance compared to emerging delivery methods, such as biomimetic foams or sprays, remains underexplored [15]. Additionally, most studies have focused on short-term outcomes, such as immediate increases in enamel mineral content, without adequately assessing long-term effects on caries prevention or overall oral health improvement. This systematic review aims to address these gaps by synthesizing existing evidence on the impact of different delivery methods of remineralization agents on dental health outcomes in pediatric patients. Specifically, the review will compare delivery methods such as varnishes, gels, foams, and sprays in terms of their effectiveness in enhancing enamel remineralization, preventing caries progression, and improving oral health indicators.

## Review

Methods

Eligibility Criteria

This systematic literature review was carried out according to the Preferred Reporting Items for Systematic Reviews and Meta-Analyses (PRISMA) criteria. The research question was: What is the relative effectiveness of different delivery methods of remineralization agents in enhancing enamel remineralization, preventing caries progression, and improving oral health outcomes in pediatric patients? The review included randomized controlled trials (RCTs), cohort studies, and case-control studies focusing on treatment outcomes with different remineralization agents on pediatric patients aged 3-15 years with early-stage dental caries or enamel demineralization (incipient or non-cavitated lesions). Eligible studies had to assess remineralization agents delivered via varnishes, gels, foams, or sprays, with outcomes related to enamel remineralization, caries prevention, or general oral health improvement published in peer-reviewed journals. Exclusion criteria included any other type of article, studies with a split-mouth design in which the investigators were studying remineralizing therapy (because there is always the risk that the agent can migrate through saliva and contaminate the control side), conducted on non-pediatric population or children with advanced caries or severe systemic diseases. Studies with a high risk of bias or significant methodological flaws and non-English language studies without verified translations were also excluded. The search was conducted based on the PICOS (Problem/Patient, Intervention/Indicator, Comparison, Outcome, and Study Design) criteria, which are presented in Table 1.

**Table 1:** Description of the PICOS elements. PICOS: Population, Intervention, Comparison, Outcome, Study Design; RCT: randomized controlled trial

Research Question:	What is the relative effectiveness of different delivery methods of remineralization agents in enhancing enamel remineralization, preventing caries progression, and improving oral health outcomes in pediatric patients?
Population:	Pediatric patients aged 3–15 years with early-stage dental caries or enamel demineralization (incipient or non-cavitated lesions)
Intervention:	Delivery methods of remineralization agents (varnishes, gels, foams, sprays)
Comparison:	Between different delivery methods.
Outcome:	Enamel remineralization, caries prevention, oral health improvement Study Design: RCTs, clinical trials, observational studies.
Study Design:	RCTs, clinical trials, observational studies.

Search Strategy

**Figure 1:**
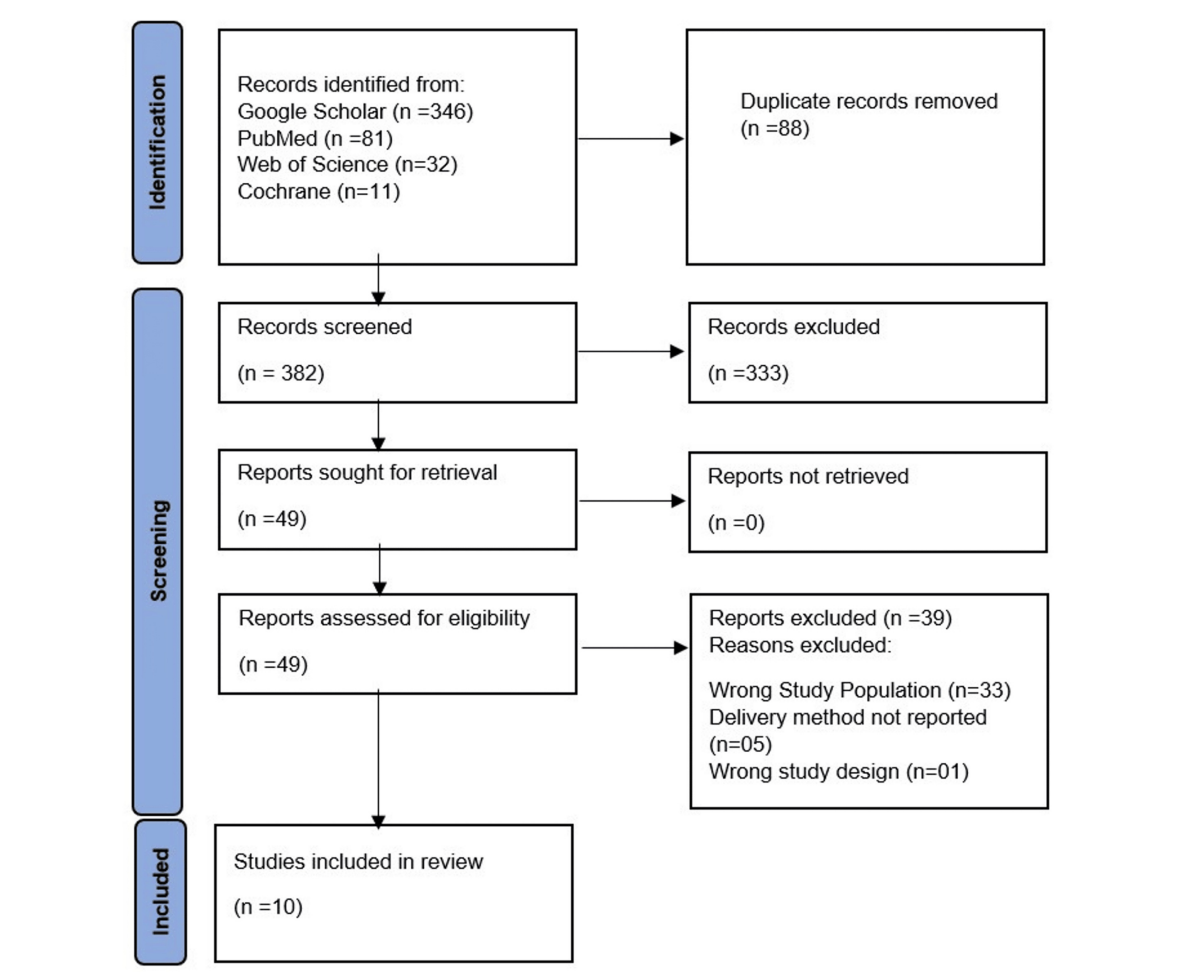
PRISMA flowchart PRISMA: Preferred Reporting Items for Systematic Reviews and Meta-Analyses

A comprehensive search was conducted in four databases: PubMed, the Cochrane Library, Google Scholar, and Web of Science. The following filters were applied to all databases: publication date within the past 10 years, English language, and studies of humans. We then searched the references cited in all the articles located to identify any additional articles. For the search, we made sure we did not miss any relevant studies by using a combination of Medical Subject Headings (MeSH) and non-MeSH terms together with Boolean operators (AND and OR) related to demineralization (tooth demineralization, white spot lesion, early/incipient caries lesion, non-cavitated enamel lesion, early carious lesion, and decalcification), pediatric dentistry (pediatric, children, child and pediatric dental health outcomes), treatment (caries prevention, tooth remineralization, cariostatic agents, fluorides, fluoride varnish, caseins, calcium phosphate, CPP-(amorphous calcium phosphate) ACP, CPP-amorphous calcium fluoride phosphate (ACFP), remineralizing agents, remineralization techniques, and minimally invasive treatment), delivery methods (varnish, foam, spray, gel, tablets, and application methods), study type (RCT, cohort, case-control, observational studies, clinical trials) and participants (human and in vivo). The search strings used in the different databases are given in Table 1. Articles published between April 2014 and November 2024 were included in the search. Discrepancies in selection were resolved through discussion or by consulting a third reviewer. A PRISMA flow diagram (Figure 1) shows the selection process.

**Table 2:** Search strings utilized across the different databases. MeSH: Medical Subject Headings; RCT: randomized controlled trial

Database	Search string (MeSH keywords/free-text terms)	Boolean operators used		Search date range
PubMed	("Pediatric"[MeSH Terms] OR "children"[MeSH Terms] OR "child") AND ("Remineralization agents" OR "fluoride varnish" OR "calcium phosphate" OR "casein phosphopeptides" OR "CPP-ACP") AND ("Dental health outcomes" OR "enamel remineralization" OR "caries prevention") AND ("varnish" OR "gel" OR "foam" OR "spray" OR "application methods") Filters: Custom range 2013-2023, Language: English. Publication date - last 10 years, Language - English, Study type - RCTs.	“AND”, “OR”	April 2014 and November 2024.	
The Cochrane Library	("Pediatric" OR "children" OR "child") AND ("Remineralization agents" OR "fluoride varnish" OR "calcium phosphate" OR "casein phosphopeptides" OR "CPP-ACP") AND ("Dental health outcomes" OR "enamel remineralization" OR "caries prevention") AND ("varnish" OR "gel" OR "foam" OR "spray" OR "application methods"). Filters: Custom range 2013-2023, Language: English. Limited to trials published in English in the last 10 years.	“AND”, “OR”	April 2014 and November 2024.	
Google Scholar	("Pediatric" OR "children" OR "child" AND "Remineralization agents" OR "fluoride varnish" OR "calcium phosphate" OR "casein phosphopeptides" OR "CPP-ACP" AND "Dental health outcomes" OR "enamel remineralization" OR "caries prevention" AND "varnish" OR "gel" OR "foam" OR "spray" OR "application methods"). Filters: Custom range 2013-2023, Language: English. Limited to trials published in English in the last 10 years.	“AND”, “OR”	April 2014 and November 2024.	
Web of Science	("Pediatric" OR "children" OR "child") AND ("Remineralization agents" OR "fluoride varnish" OR "calcium phosphate" OR "casein phosphopeptides" OR "CPP-ACP") AND TS=("Dental health outcomes" OR "enamel remineralization" OR "caries prevention") AND TS=("varnish" OR "gel" OR "foam" OR "spray" OR "application methods") Filters: Custom range 2013-2023, Language: English. Last 10 years, Language: English, Document types: Articles.	“AND”, “OR”	April 2014 and November 2024.	

Data Collection and Analysis

Data extraction and assessment of bias: Two independent reviewers conducted the same search independently and screened titles and abstracts for relevance, followed by a full-text review of eligible studies. Data were extracted using a standardized form that included study characteristics (author, year, location, design), population details (age, health status), interventions (type and delivery method of remineralization agents), comparators, and reported outcomes. Discrepancies in selection were resolved through discussion or by consulting a third reviewer.

Data Synthesis and Statistical Analysis

A narrative synthesis was performed to summarize findings, supported by tables and figures. Data was visualized using Microsoft Excel (Microsoft Corporation, Redmond, Washington, United States), and separate sheets were maintained and collaborated on by the reviewers during data extraction.

Quality Assessment

**Figure 2:**
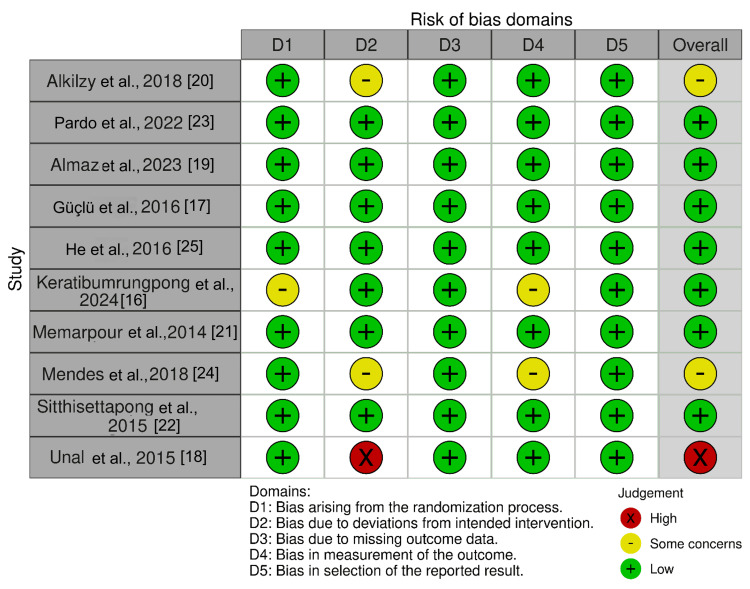
Risk of bias assessment using Cochrane ROB tool. References: [16-25]

The methodological quality of the 10 included studies was evaluated independently by two reviewers using the Cochrane ROB-2 tool as shown in Figure 2. Studies were categorized as low, moderate, or high risk of bias based on factors such as randomization, blinding, and completeness of outcome data. Any disagreements were resolved by consensus.

Out of the 10 studies, eight exhibited an overall low risk of bias, with most domains (D1-D5) judged favorably. However, concerns about randomization (D1) or deviations from the intended intervention (D2) were noted in studies by Keratibumrungpong et al. [16] and Mendes et al. [24]. Notably, Unal and Oztas's study [18] had a high risk of bias, particularly in deviations from intervention (D2) and overall assessment. This variation may stem from inadequate blinding or intervention inconsistencies.

Results

Ten RCTs were included in the analysis, conducted across diverse countries, including Germany [20], Italy [23], Turkey [17-19], China [25], Thailand [16,22], Iran [21], and Brazil [24], and encompassing a total sample size of 743 participants. Table 3 summarizes the characteristics of the studies. Sample sizes ranged from 21 to 240 participants, with interventions tailored to specific populations. Alkilzy et al. [20], Pardo et al. [23], and Almaz et al. [19] explored fluoride varnishes combined with innovative agents (e.g., self-assembling peptides, biorepair pastes). Güçlü et al. [16], He et al. [25], and Memarpour [21] targeted white spot lesions using fluoride, CPP-ACP, or a combination. Keratibumrungpong et al. [16] and Mendes et al. [24] compared fluoride gels and pastes against control groups. Sitthisettapong et al. [22] emphasized daily CPP-ACP application for high caries-risk preschool children, while Unal and Oztas [18] investigated fissure sealants with or without ozone.

**Table 3:** Characteristics of the included studies RCT: randomized controlled trial; ICDAS: International Caries Detection and Assessment System; MIH: molar-incisor hypomineralization; DMFS: decayed, missing, and filled surfaces; TCP: tricalcium phosphate; NaF: sodium fluoride; CPP-ACP: casein phosphopeptide-amorphous calcium phosphate; WSL: white spot lesion; FV: fluoride varnish; PFS: pit and fissure sealant

Study	Country	Study Design	Sample Size	Age Range of Subjects	Population	Intervention	Comparison
Alkilzy et al., 2018 [20]	Germany	RCT	70 participants (35 in the test group and 35 in the control group)	Mean age: 10.0 ± 2.7 years	Children (>5 years old) with visible and accessible active early occlusal caries (ICDAS 1–3) on erupting permanent molars; high to moderate caries risk	Self-assembling peptide P11-4 (Curodont Repair) + fluoride varnish (22,600 ppm, Duraphat)	Fluoride varnish alone
Pardo et al., 2022 [23]	Italy	RCT	25 participants (50 teeth)	Mean age: 8.6 ± 1.22 years (range: 6–10 years)	Children with MIH; good general health; no systemic diseases or orthodontic therapy	Biorepair® Desensitizing Enamel-Repair Shock Treatment paste applied using a bite appliance	No treatment (contralateral MIH teeth in the same individual served as control)
Almaz et al., 2023 [19]	Turkey	RCT	140 teeth from 48 children (12 children per group, 35 teeth per group)	6–7 years (Mean age: 6.8 ± 0.7 years)	Healthy, high caries risk children (DMFS > 8) with newly erupted permanent first molars	Duraphat Varnish (5% NaF), Clinpro™ White Varnish (5% NaF with TCP), Embrace™ Varnish (5% NaF with CXP), MI Varnish (5% NaF with CPP-ACP)	Comparison between four varnish types (no placebo or control)
Güçlü et al., 2016 [17]	Turkey	RCT	21 healthy children (from an initial 30 participants)	8-15 years	Healthy children referred for treatment of WSL	Fluoride varnish (FV), 5% sodium fluoride; CPP-ACP paste (GC Tooth Mousse); CPP-ACP + Fluoride varnish (CPP-ACP-FV)	Control group (no treatment or intervention)
He et al., 2016 [25]	China	RCT	240 patients (597 teeth)	12-20yrs	Patients who recently finished orthodontic treatment, with at least one maxillary anterior tooth with a WSL	Fluoride varnish (Duraphat, 5% sodium fluoride) or fluoride film (Sheer, 5% acidulated sodium fluoride)	Placebo (fluoride-free deliquescent toothpaste)
Keratibumrungpong et al., 2024 [16]	Thailand	RCT	29 participants (9 males, 16 females)	12-15 years old	Healthy students identified as high caries risk	Placebo group: Non-fluoride gel (paint-on application); Test group I: 1.23% APF gel (paint-on application); Test group II: 1.23% APF gel (tray application)	Placebo (non-fluoride gel)
Memarpour et al., 2014 [21]	Iran	RCT	140 children	12 to 36 months (mean age: 21.20 ± 6.76 months)	Children aged 12-36 months, with at least 4 erupted maxillary primary incisors, present WSL but no cavitated caries.	Fluoride varnish (5% sodium fluoride) (Group 3)	-Control group (no intervention) (Group 1) -Oral hygiene and diet counseling (Group 2) -Oral hygiene and tooth mousse (CPP-ACP) (Group 4)
Mendes et al., 2018 [24]	Brazil	RCT	45 children	5 to 13 years	Children with active white spot lesions on permanent incisors and canines	G1: Placebo paste, G2: Fluoride gel (1.23% acidulated phosphate fluoride), G3: CPP-ACP (Recaldent), G4: CPP-ACP + fluoride (Recaldent Plus)	G1: Placebo paste
Sitthisettapong et al., 2015 [22]	Thailand	RCT	103 children enrolled, with 79 completing the study	2.5 to 3.5 years (mean age: 37.51 ± 2.93 months)	Healthy preschool children with high caries risk	10% w/v CPP-ACP (Tooth Mousse®) daily	Placebo paste with identical packaging, color, and taste
Unal and Oztas, 2015 [18]	Turkey	RCT	60 participants (29 girls, 31 boys)	7-9 years (mean age = 7.6 ± SD not specified)	Children with initial pit and fissure caries on enamel (non-cavitated) on fully erupted mandibular first molars.	Fissure Sealants (PFS): Aegis ACP, Fuji Triage, Helioseal with or without ozone application.	Control group (no ozone application)

The participants were primarily children, with ages ranging from 12 months to 20 years, depending on the study. All studies focused on pediatric populations with specific dental conditions such as early occlusal caries, molar-incisor hypomineralization (MIH), white spot lesions (WSLs), or high caries risk. Male predominance was noted in some studies, though gender distribution varied. The mean ages of participants spanned from 21 months to 15 years, with the highest mean age observed in orthodontic patients with WSLs. As mentioned earlier, interventions varied widely across studies. Most employed remineralization agents like fluoride varnishes, self-assembling peptides, or CPP-ACP formulations, with some studies comparing these agents or assessing different application methods. Comparisons included control groups receiving no treatment, placebos, or alternative preventive interventions. Delivery methods included varnishes, gels, films, pastes, and even fissure sealants combined with or without ozone.

The patient populations showed significant variation in health conditions and dental needs. Inclusion criteria ranged from children with early caries and non-cavitated lesions to those undergoing orthodontic treatment. All studies stratified participants into intervention and control groups, aiming to evaluate the efficacy of novel and conventional remineralization methods in improving oral health outcomes in pediatric populations. Table 4 summarizes the delivery methods and remineralization agents used, along with their effectiveness.

**Table 4:** Summary of delivery methods, remineralization agents, and their effectiveness VAS: Visual Analog Scale; ICDAS: International Caries Detection and Assessment System; MIH: molar-incisor hypomineralization; TNI: Treatment Need Index; NaF: sodium fluoride; CPP-ACP: casein phosphopeptide-amorphous calcium phosphate; CXP: calcium exchange phosphate; SAI: Schiff Air Index; TCP: tri-calcium phosphate; FV: fluoride varnish; DMFT: decayed, missing, filled teeth; QLF: quantitative light-induced fluorescence

Study	Delivery Method	Remineralization agent used		Application Frequency	Application Duration	Assessment Method	Effectiveness
Alkilzy et al., 2018 [20]	Peptide applied once at baseline; fluoride varnish applied at baseline and 3-month follow-up	Self-assembling peptide P11-4; fluoride varnish	Baseline for both interventions; additional fluoride varnish at 3 months		Not specified; applied per manufacturer’s instructions.	Laser fluorescence readings (Diagnodent), VAS, ICDAS, Nyvad criteria.	Significant decrease in laser fluorescence readings and VAS scores in the test group compared to control at 3 and 6 months
Pardo et al., 2022 [23]	Bite appliance	Zinc-hydroxyapatite	Weekly, for 1 week per month		10 minutes per session	Plaque Control Record (PCR), Bleeding Index (BI), MIH (MIH-TNI), SAI	Significant improvement in MIH-TNI and SAI scores in the test group compared to the control group at T9 (p < 0.05).
Almaz et al., 2023 [19]	Varnish (Duraphat, Clinpro™ White, Embrace™, MI)	NaF; TCP; CPP-ACP, CXP	Applied at baseline, 1 month, and 3 months		Not specified (general clinical practice for varnish application)	Laser Fluorescence (LF) measurements using DIAGNOdent pen 2190 (KaVo) at baseline, 1 month, 3 months, and 6 months	NaF Varnish, NaF with TCP Varnish, and NaF with CPP-ACP Varnish showed significant remineralization at 3 and 6 months - NaF with CXP Varnish showed the least remineralization effect at 3 and 6 months compared to others, but was still effective - NaF with CPP-ACP Varnish exhibited the greatest remineralization at 6 months.
Güçlü et al., 2016 [17]	FV, CPP-ACP paste, or both in combination (CPP-ACP-FV)	Fluoride varnish (5% sodium fluoride), CPP-ACP paste (GC Tooth Mousse)	Weekly consultations for 12 weeks		Fluoride varnish: 5 treatments (weeks 1-4 and week 12) CPP-ACP paste: Morning and evening after brushing (for 12 weeks)	Visual assessment (using criteria by Ekstrand et al. [26]), Laser fluorescence (LF) measurements (DIAGNOdent)	Significant improvement in WSLs visual appearance (regression or stabilization) in FV, CPP-ACP, and CPP-ACP-FV groups; no improvement in the control group. Laser fluorescence showed significant remineralization in all treatment groups (p < 0.001).
He et al., 2016 [25]	Fluoride varnish, fluoride film, placebo (fluoride-free toothpaste)	Fluoride (NaF in varnish, acidulated NaF in film)	Once a month		No specific duration mentioned, treatment was applied during monthly visits	QLF imaging, assessing DF (%), area (mm²), and DQ (mm² × %)	Fluoride varnish and fluoride film groups showed significant improvements in demineralization metrics (DQ, DF, area) after 3 and 6 months. The control group showed less improvement.
Keratibumrungpong et al., 2024 [16]	1. Paint-on application, 2. Tray application	1. Placebo: Non-fluoride gel, 2. Test group I: 1.23% APF gel, 3. Test group II: 1.23% APF gel	Single application (1-minute for paint-on, 4 minutes for tray)		1 minute (paint-on application) 4 minutes (tray application)	QLF-digital (D)	This technique could be effectively used as an adjunctive method for fluoride applica tion and could be advantageous for younger patients.
Memarpour et al., 2014 [21]	Fluoride varnish (Group 3), Tooth mousse (CPP-ACP) (Group 4)	Fluoride (5% sodium fluoride) (Group 3) CPP-ACP (tooth mousse) (Group 4).	Fluoride varnish applied every 4 months (Group 3), Tooth mousse applied twice daily (Group 4)		Fluoride varnish: 1 minute per session (Group 3), Tooth mousse: 3 minutes per session (Group 4)	Visual inspection and measurement of WSL area. DMFT index.	Significant reduction in WSL in Group 4 (CPP-ACP) and Group 3 (fluoride varnish) compared to the control group after 12 months (Group 1). Group 3 (fluoride varnish) showed a 51% decrease in WSL after 12 months, and Group 4 (tooth mousse) showed a 63% decrease. Group 2 (oral hygiene counseling) showed only a 10% decrease in WSL.
Mendes et al., 2018 [24]	G1: Paste, G2: Gel, G3: Paste, G4: Paste	G1: Placebo, G2: Fluoride, G3: CPP-ACP, G4: CPP-ACP + Fluoride	G1, G2, G3, G4: Twice, one-week interval		60 seconds	Lesion intensity measured with DIAGNOdent Pen (fluorescence intensity)	G2 (fluoride gel) and G4 (CPP-ACP + fluoride) showed significant decrease in lesion intensity after 30 days, with G4 maintaining the lowest intensity at 90 days. G1 and G2 did not show sustained benefits by 90 days. G3 (CPP-ACP) showed improvement at 8 days but had the greatest demineralization by 90 days.
Sitthisettapong et al., 2015 [22]	Paste (applied using a disposable cotton-tipped applicator)	CPP-ACP	Daily application (after lunch and toothbrushing with fluoridated toothpaste)		After application, children were instructed to keep the paste in the mouth for 30 minutes, avoiding swallowing or expectoration	QLF	Significant remineralization of enamel lesions in the CPP-ACP group compared to the placebo group over 1 year (based on ΔQ, fluorescence loss, and lesion area measurements).
Unal and Oztas, 2015 [18 ]	Varnish (Aegis ACP), Glass ionomer (Fuji Triage), Resin-based (Helioseal)	Fluoride-based (Aegis ACP), Glass ionomer (Fuji Triage), Resin (Helioseal)	Once (with follow-up at 1st, 3rd, 6th, 9th, and 12th months)		Not specifically mentioned for the sealant application, ozone treatment: 40 seconds application, 10 seconds vacuum	DIAGNOdent measurements (pre-treatment and after 12 months), clinical retention assessments	Significant changes in DIAGNOdent values for groups 1 (Aegis ACP) and 2 (Fuji Triage) (p<0.05), no significant change in group 3 (Helioseal). Full retention did not significantly differ across groups (p>0.05) except for Fuji Triage (p<0.05) after 12 months.

The Grading of Recommendations Assessment Development and Evaluation (GRADE) approach was utilized to determine the certainty of the evidence in this systematic review. The certainty of the evidence was evaluated based on three domains: Studies included, Number of participants, and Effectiveness. It was classified as very low, low, moderate, or high certainty of evidence. Our results are summarized in Table 5 along with the GRADE assessment of each outcome. 

**Table 5:** The Grading of Recommendations Assessment Development and Evaluation (GRADE) CPP-ACP: casein phosphopeptide-amorphous calcium phosphate

Outcome	Studies Included	No. of participants	Effectiveness	GRADE assessment
Enamel Remineralization	10 RCTs	743	All delivery methods showed improvement; varnishes had the highest effect, followed by CPP-ACP gels.	Moderate (heterogeneity in protocols and sample sizes)
Caries Prevention	7 RCTs	520	Varnishes and CPP-ACP gels significantly reduced caries progression compared to control groups.	High (consistent results across studies).
Patient Compliance	6 RCTs	430	Foams and sprays improved compliance due to ease of use, but had lower efficacy compared to varnishes or gels.	Low (limited data, high variability in measurement)
Long-Term Outcomes	3 RCTs	210	Limited evidence; varnishes showed sustained benefits over 12 months.	Very Low (insufficient long-term data)

Discussion

Comparison of Delivery Methods

The studies analyzed in this systematic review demonstrate that the mode of delivery significantly influences the effectiveness, patient compliance, and overall practicality of remineralization agents for pediatric dental care. The four delivery methods reviewed, varnishes, gels, foams, and sprays, each present unique advantages and limitations. A summary of the comparison can be seen in Table 6. By comparing these methods, this section highlights their strengths and weaknesses to guide clinical decision-making.

**Table 6:** Comparison of delivery methods

Delivery Method	Benefits	Pitfalls
Varnishes	High fluoride concentration enhances remineralization. Prolonged enamel contact improves efficacy. Suitable for both primary and permanent teeth.	Requires professional application. Limited patient autonomy for reapplication.
Gels	High remineralization potential due to concentrated application. Effective for treating widespread lesions.	Tray placement may cause discomfort or gagging in children. Requires significant patient cooperation, limiting use in anxious or very young patients.
Foams	Minimally invasive and quick to apply. Suitable for anxious patients.	Limited clinical evidence on long-term effectiveness. May not deliver as high concentrations as varnishes or gels.
Sprays	Easy to apply, with potential for self-administration. Non-invasive and well-accepted by children.	Uneven distribution limits efficacy. Less durable enamel contact time.

Varnishes: Fluoride varnishes, such as Duraphat and Clinpro, are a mainstay in caries prevention and remineralization due to their high fluoride content and ability to provide prolonged contact with enamel [14,15]. Their semi-viscous consistency allows easy application, even in uncooperative pediatric patients, and their rapid drying minimizes ingestion risks [19]. Studies, including Alkilzy et al. [20] and Memarpour et al. [21], consistently demonstrated that fluoride varnishes significantly reduced caries progression and enhanced remineralization of early lesions. However, their efficacy is limited by the application frequency and the dependence on professional administration, which can increase costs and reduce accessibility in underserved areas [15].

Gels: Gels such as 1.23% APF gel and CPP-ACP formulations often require tray applications, which provide a controlled environment for high-concentration agents to interact with the enamel [22]. Their advantages include the ability to deliver higher doses of fluoride or other remineralizing agents over a short period [23]. However, as observed in studies like Mendes et al. [24], their use in pediatric populations is complicated by discomfort during tray placement and challenges with patient cooperation, particularly among younger children.

Foams: Emerging as a novel delivery system, foams are lightweight and easy to distribute, making them particularly suitable for patients with behavioral challenges [27]. While limited evidence is available, foams are hypothesized to enhance compliance due to their simplicity and minimal invasiveness. Their ability to penetrate deep lesions is still under investigation. Foams show promise for routine preventive care but require further research to validate their efficacy compared to more established methods.

Sprays: Sprays offer a highly convenient and patient-friendly approach. They are ideal for rapid application and can be self-administered, providing an advantage in settings where professional application is not feasible. However, studies like He et al. suggest that sprays may suffer from uneven distribution and lower adherence to enamel surfaces, potentially reducing their remineralization capacity [25].

Advantages of Combining Delivery Methods

A notable finding from the review was the synergistic effect observed in combining delivery methods or agents. For example, Alkilzy et al. demonstrated improved outcomes when fluoride varnish was paired with self-assembling peptides [20]. Such combinations allow for the strengths of one delivery method to complement the limitations of another. Future clinical protocols could consider hybrid approaches to optimize treatment efficacy.

High Risk vs. Low Risk Caries Patients and Choice of Delivery Method

The choice of an ideal delivery method should also account for the patient's caries risk. High-caries-risk patients, who often require intensive and frequent remineralization therapy, benefit from delivery methods that ensure prolonged agent retention, such as fluoride varnishes or CPP-ACP gels [28]. These methods allow sustained release of active ingredients, providing long-lasting protection against demineralization. On the other hand, low-caries-risk patients might achieve sufficient preventive outcomes with less intensive methods, such as sprays or foams, which are easier to apply but may require more frequent applications to maintain effectiveness. Stratifying delivery methods based on caries risk enables clinicians to tailor treatments to patient needs, optimizing resource allocation while maintaining clinical efficacy.

Ideal Delivery Method and Proper Isolation

The application method significantly influences remineralization agents' effectiveness, particularly in the context of proper isolation techniques like rubber dam usage. Rubber dam isolation creates a dry, uncontaminated field, enhancing the adhesion and penetration of agents like FVs, CPP-ACP pastes, and self-assembling peptides into enamel microstructures. Studies have demonstrated that the absence of moisture or saliva contamination during application increases the efficacy of these agents by facilitating a more uniform layer deposition and prolonged agent retention on the tooth surface [29]. Furthermore, methods such as varnishes, which adhere well in dry conditions, often exhibit superior outcomes when combined with proper isolation. The use of rubber dams is particularly crucial for younger patients, where maintaining a saliva-free environment can be challenging.

Behavioral and Psychological Considerations

One key challenge in pediatric dental care is ensuring patient compliance, which varies significantly with the invasiveness and duration of treatment. Varnishes and sprays are better tolerated by anxious children or those with shorter attention spans, while gels, requiring extended contact time, may be less acceptable [30]. The choice of delivery method should balance efficacy with patient comfort to improve adherence and treatment outcomes.

How Patient Behavior Influences Delivery Method Used

As outlined in Table 6, patient behavior plays a pivotal role in determining the feasibility and success of various delivery methods. Cooperative patients, who can remain still and follow instructions, are suitable candidates for methods requiring precision, such as tray-based gel applications or fissure sealants combined with remineralization agents. Potentially uncooperative patients, often younger children or those with anxiety, may respond better to faster and less invasive methods like fluoride varnishes or sprays, which can be applied quickly with minimal discomfort. For completely uncooperative patients, such as those with special needs or severe dental fear, methods like varnishes combined with behavior management techniques or even sedation may be necessary. Understanding patient behavior not only aids in selecting the most effective delivery method but also enhances patient comfort and compliance during dental procedures.

Clinical Implications and Recommendations

The clinical decision on the optimal delivery method should consider the patient’s age, caries risk level, and behavioral profile. High-risk populations may benefit from professional-grade varnishes or intensive gel applications to achieve maximum remineralization. Younger or behaviorally challenging patients may respond better to foams or sprays due to their ease of application. Additionally, accessibility and cost-effectiveness play a significant role, particularly in low-resource settings. Spray and foam methods, given their potential for self-application, could be more feasible alternatives in such contexts. However, the lower efficacy observed with these methods suggests the need for further optimization or combination with adjunctive therapies.

Several limitations in the existing literature hinder the ability to make definitive recommendations about the best delivery methods for remineralization agents. Short follow-up periods in many studies limit the understanding of long-term efficacy in preventing caries and maintaining enamel health. Furthermore, significant heterogeneity exists across studies regarding application protocols, including differences in agent concentrations and frequencies of use, making direct comparisons challenging. Emerging delivery methods such as foams and sprays have also received limited attention, with few robust trials exploring their potential advantages or optimal application in pediatric populations.

Addressing these gaps requires future research to focus on long-term studies that assess sustained caries prevention and enamel durability across various delivery methods. Conducting head-to-head trials under standardized conditions could provide clearer clinical guidelines by enabling more reliable comparisons. Moreover, examining the behavioral aspects of these interventions such as strategies to enhance patient compliance will be crucial for improving their real-world effectiveness, particularly among pediatric patients.

## Conclusions

This systematic review highlights the significant impact of delivery methods on the effectiveness of remineralization therapies in pediatric dental care. While varnishes continue to be the gold stander for high-risk caries populations, sprays and foams offer valuable alternatives, particularly for children with behavioral challenges. Ultimately, personalizing remineralization treatments to the specific needs of each patient is key to maximizing the success of these therapies and improving long-term pediatric dental health outcomes.
